# NQO1 potentiates apoptosis evasion and upregulates XIAP via inhibiting proteasome-mediated degradation SIRT6 in hepatocellular carcinoma

**DOI:** 10.1186/s12964-019-0491-7

**Published:** 2019-12-16

**Authors:** Hong-Zhong Zhou, Han-Qing Zeng, Ding Yuan, Ji-Hua Ren, Sheng-Tao Cheng, Hai-Bo Yu, Fang Ren, Qing Wang, Yi-Ping Qin, Ai-Long Huang, Juan Chen

**Affiliations:** 1 The Key Laboratory of Molecular Biology of Infectious Diseases designated by the Chinese Ministry of Education, Institute for Viral Hepatitis, Department of Infectious Diseases, The Second Affiliated Hospital, Chongqing Medical University, Room 617, College of Life Sciences Building, 1 YiXueYuan Road, YuZhong District, Chongqing, 400016 China; 2 Department of Hematology, The Second Affiliated Hospital, Chongqing Medical University, Chongqing, China; 3 Department of Neurosurgery, The Fifth People’s Hospital of Chongqing, Chongqing, China

**Keywords:** NQO1, SIRT6, XIAP, Hepatocellular carcinoma, Apoptosis

## Abstract

**Background:**

Our previous study has demonstrated that NAD(P)H: quinone oxidoreductase 1 (NQO1) is significantly upregulated in human liver cancer where it potentiates the apoptosis evasion of liver cancer cell. However, the underlying mechanisms of the oncogenic function of NQO1 in HCC have not been fully elucidated.

**Methods:**

Expression of NQO1, SIRT6, AKT and X-linked inhibitor of apoptosis protein (XIAP) protein were measured by western blotting and immunohistochemistry. Additionally, the interaction between NQO1 and potential proteins were determined by immunoprecipitation assays. Furthermore, the effect of NQO1 and SIRT6 on tumor growth was determined in cell model and orthotopic tumor implantation model.

**Results:**

We found that NQO1 overexpression in HCC enhanced SIRT6 protein stability via inhibiting ubiquitin-mediated 26S proteasome degradation. High level of SIRT6 reduced acetylation of AKT which resulted in increased phosphorylation and activity of AKT. Activated AKT subsequently phosphorylated anti-apoptotic protein XIAP at Ser87 which determined its protein stability. Reintroduction of SIRT6 or AKT efficiently rescued NQO1 knock-out-mediated inhibition of growth and induction of apoptosis. In orthotopic mouse model, NQO1 knock-out inhibited tumor growth and induced apoptosis while this effect was effectively rescued by SIRT6 overexpression or MG132 treatment partially.

**Conclusions:**

Collectively, these results reveal an oncogenic function of NQO1 in sustaining HCC cell proliferation through SIRT6/AKT/XIAP signaling pathway.

## Background

NAD(P)H: quinone oxidoreductase 1 (NQO1) is originally identified as an antioxidant enzyme which catalyzes the two-electron oxidoreduction to generate an unstable hydroquinone [1]. Recent studies have showed that NQO1 is upregulated in many human cancers, including Cholangiocarcinoma [2], non-small cell lung cancer [3], uterine cervical cancer [4], prostate cancer [5, 6] and pancreatic cancer [7]. In breast, lung cancer and uterine cervical cancer, high-level NQO1 expression was closely associated with poor differentiation, late clinical stage and lymph node metastasis. Patients with high-level expression of NQO1 had a shorter disease-free survival and overall survival rate than those with low NQO1 expression in cell lung cancer [3]. Previously, we have reported that NQO1 is generally increased in HCCs where its high expression level is related with poor patient survival rate. NQO1 potentiates the apoptosis evasion of liver cancer cell through upregulating X-linked inhibitor of apoptosis protein (XIAP) protein stability [8]. However, the molecular mechanisms by which NQO1 exerts pro-tumorigenic function need to further elucidated.

Sirtuin 6 (SIRT6), a member of the sirtuin family, is NAD^+^-dependent deacetylases with important roles in glucose homeostasis, maintenance of genome stability and cancers development [9–11]. Because of its complex and opposite functional roles, SIRT6 is considered a two-edged sword in carcinogenesis [12]. In particular, SIRT6 deacetylates lysine 56 of histone H3 (H3K56) and lysine 9 of histone H3 (H3K9), which has a duty for reduced chromatin accessibility to transcription factors. Interestingly, it was found that acetylation of H3K56 is increased in multiple types of cancer including, liver, breast, thyroid and colon cancer [13, 14]. Importantly, SIRT6 plays a tumor suppressor role in the maintenance of cancer [15]. On the other hand, SIRT6 is overexpressed in some cancers, such as squamous cell carcinoma (SCC), breast cancer, ovarian cancer and multiple myeloma, suggesting it possesses oncogenic activity [16–19]. SIRT6 overexpression increases cancer cell proliferation by directly modulating oncogenic proteins or the acetylation of H3K9 and H3K56 of oncogene promoter dependent on its deacetylase activity [13, 14]. In HCC, SIRT6 potentiates apoptosis evasion of HCC cells via chromatin remodeling. Mechanistically, SIRT6 induces H3K9 deacetylation that blocks Bax transcription, and enhances E2F-1 and p53 chromatin accessibility [20]. 3′,5′-Cyclic adenosine monophosphate (cAMP), AKT, miR-34a and miR-122 are involved in the regulation of transactivation and expression of SIRT6 [16, 18, 21, 22]. However, the investigations on the stability of SIRT6 are limited.

Excepting its oxidoreductase activity, NQO1 has been reported to stabilize many proteins, including p33 [23], p53 [24], p73 [25], eIF4GI [26] and c-Fos [27], by inhibiting their proteasomal degradation. In this study, we demonstrated that NQO1 interacts physically with SIRT6, stabilizes the protein and prevents it from ubiquitin-dependent 26S proteasomal degradation. Consequently, SIRT6 deacetylated AKT to promote its phosphorylation and activation, thus leading to increased XIAP phosphorylation and protein stability. Taken together, our results strongly suggested an important role of NQO1-induced SIRT6 stability in tumor biology of HCC.

## Methods

### Cell lines

Huh-7 and PLC/PRF/5 were obtained from the Heath Science Research Resource Bank (HSRRB) and American Type Culture Collection (ATCC), respectively. The two cell lines were routinely maintained in Dulbecco’s modified Eagle’s medium containing 10% fetal bovine serum (Gibco-BRL) and penicillin/streptomycin, and cultured at 37 °C with 5% CO_2_ in a humidified incubator. Cell lines were authenticated by short tandem repeat analysis technology (STR), and examined negative for mycoplasma.

### Antibodies, plasmids and chemicals

The pcDNA3-Xiap-Myc (#11833) plasmid was purchased from Addgene (Cambridge, MA). The plasmids of Myc-DDK-tagged-SIRT6 (RC202833) and pCMV6-NQO1 (SC119599) were obtained from OriGene Technologies (Rockville, MD). Rabbit anti-AKT (#4691), rabbit anti-phospho-AKT (#4060), mouse anti-ubiquitin (#3936), rabbit anti-acetylated-Lysine (#9441), rabbit anti-SIRT1 (#9475), rabbit anti-SIRT2 (#12650), rabbit anti-SIRT3 (#2627), rabbit anti-SIRT5 (#8782) and rabbit anti-Ki67 (#9027) were bought from CST (Danvers, MA). Rabbit anti-SIRT6 (NB100–2522) was obtained from Novus Biologicals (Colorado, USA). Rabbit anti-SIRT7 (S5947) was purchased from Sigma (Saint Louis, MO). Rabbit anti-26S (14748–1-AP) were obtained from Proteintech. Mouse anti-SIRT4 (SC-135798) was purchased from Santa Cruz. MG132 (S2619), MK-2206 2HCL (S1078) and PYR-41 (S7129) were obtained from Selleck Chemicals (Houston, USA). Cycloheximide (a protein synthesis inhibitor in eukaryotes [28]) was purchased from Calbiochem (Merck). Dicoumarol (the most potent inhibitor of NQO1 [29]) was purchased from MERCK (287897).

### Coimmunoprecipitation assay and western blotting analysis

Coimmunoprecipitation assays were performed as described previously [20]. Briefly, cell lysates were precipitated in an immunoprecipitation assay buffer at 4 °C overnight with appropriated antibodies (anti-XIAP, anti-SIRT6, anti-AKT, anti-NQO1, anti-26S). Proteins were separated by 8–12% SDS-PAGE, immunocomplexes then were subjected to western blotting analysis.

### Detection of proliferation and apoptotic cells

To examine cell proliferation rate, the same number of cells were seeded into 6-well plates. Cell viability as well as cell number were assessed using trypan blue exclusion assay. Apoptotic process was detected according to the manufacturer’s instructions. Then the cells were analyzed by flow cytometry immediately.

### Immunohistochemistry (IHC)

Immunohistochemistry for target proteins were executed on paraffin sections using appreciated antibodies (anti-cleaved PARP, 1:100; anti-XIAP, 1:50; anti-NQO1,1:50; anti-SIRT6, 1:100). IHC was carried out as described by Sun et.al [30]. In brief, staining was assessed based on the percentage of positively stained cells. And the staining intensity was analyzed by Image-Pro Plus 6.0 software.

### Animal studies

For tumor formation in nude mice, indicated cells (1 × 10^6^) were harvested in 30 μl medium (DMEM: Matrigel = 1:1) and were injected into the left lobe of liver of 5 or 6-week-old male nude mice. Thirty-two nude mice were divided into four groups randomly (*n* = 8). Nude mice received injections with or without MG132 (0.05 mg/kg, intraperitoneally, daily) for 30 days starting 5 days after the injection of indicated cells.

Histopathological examination was executed using IHC staining as previously described. All of the procedures for handling of animals abided by the guidelines of Chongqing Medical University Animal Care Committee (reference number: 2019002), and were approved by the institutional animal research ethical committee.

### Statistical analysis

All statistical analyses were conducted using GraphPad Prism5 (GraphPad) software unless otherwise indicated. Data were shown as mean ± S.E.M. from three independent experiments. For comparisons, a two tailed student’s t test, Wilcoxon signed-rank test, Mann-Whitney test were performed as indicated. *p* value < 0.05 was considered significant statistically.

## Results

### NQO1 regulates XIAP phosphorylation via AKT activation

**Fig. 1:**
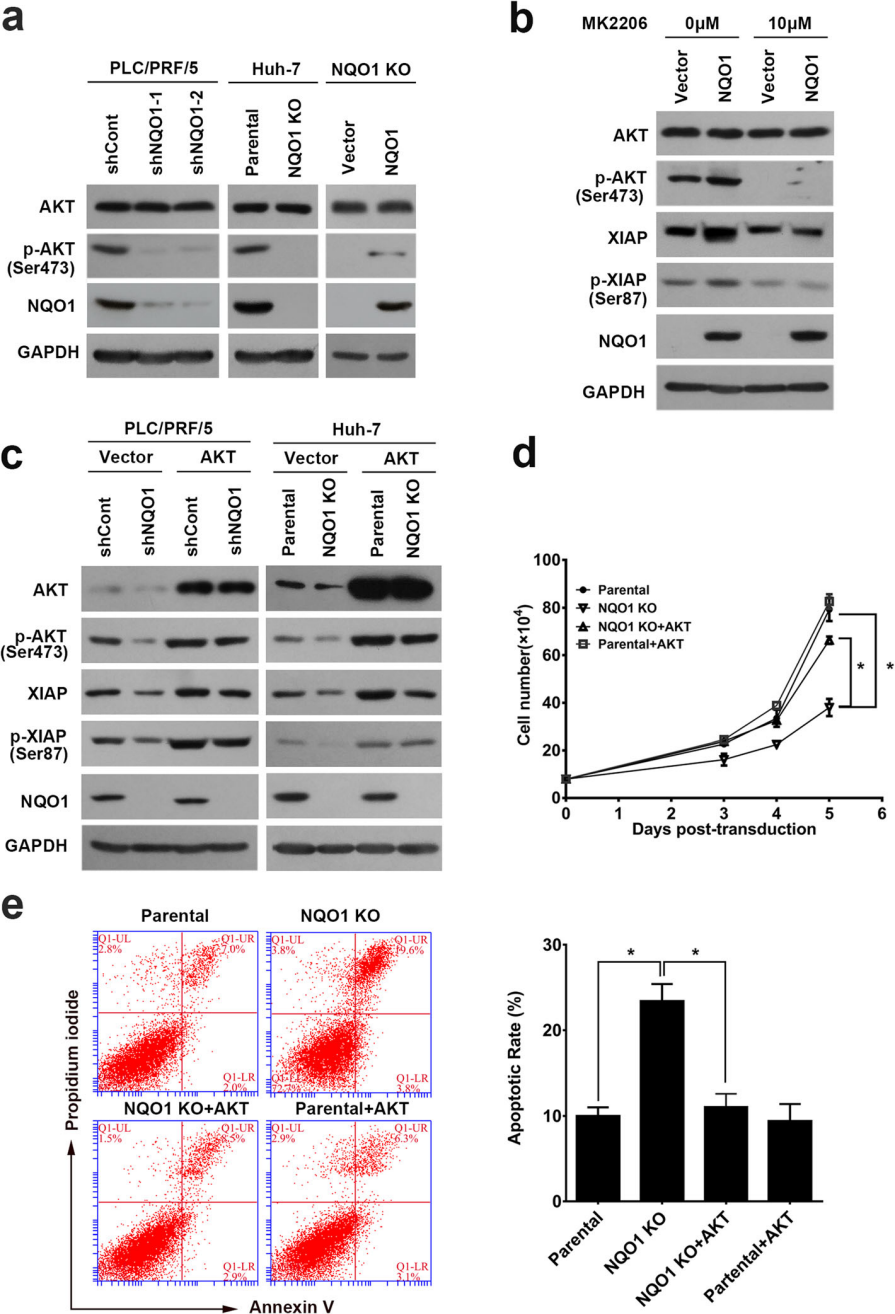
NQO1 increases XIAP phosphorylation via AKT activation. **a** Immunoblotting analysis for AKT and phosphor-AKT (pSer473) in NQO1 knock-down/knock-out cells or NQO1 knock-out cells transfection of vector expressing NQO1. **b** Immunoblotting analysis for AKT, phosphor-AKT (pSer473), XIAP, and phosphor-XIAP (pSer87) in NQO1 knock-out cells. NQO1 cells were transfected with plasmid expressing NQO1 and then treated with AKT inhibitor MK2206 (10 μM) for 24 h. **c** Immunoblotting analysis for AKT, phosphor-AKT (pSer473), XIAP and phosphor-XIAP (pSer87) in NQO1 knock-down/knock-out cells transfected with AKT or empty vector. **d**-**e** Trypan blue exclusion assay (**d**) and flow cytometry (**e**) were performed to analyze the NQO1-depleted cells transfected with AKT. Data are mean ± SEM of *n* = 3 independent experiments

As reported in our previous study [8], NQO1 was generally upregulated in HCC, which was associated with poor prognosis. NQO1 inhibitor or NQO1 knock-down/knock-out reduced the level of XIAP protein via decreasing the phosphorylation status of XIAP at ser87. It has been reported that XIAP was phosphorylated and stabilized by AKT at Ser87 [31–34], we then analyzed whether NQO1 regulated AKT expression and activation. Interestingly, knock-down/knock-out of NQO1 reduced the phosphorylation status of AKT without affecting total expression of AKT, whereas NQO1 overexpression increased the level of phosphorylated AKT (Fig. 1a). Importantly, AKT inhibitor (MK2206) remarkably blocked NQO1-induced XIAP expression and phosphorylation (Fig. 1b). Introduction of AKT which increased the level of phosphorylated AKT markedly rescued NQO1 silencing-induced decrease of XIAP expression and phosphorylation (Fig. 1c). Moreover, introduction of AKT rescued NQO1 silencing-inducing growth inhibition and apoptosis in HCC cells (Fig. 1d-e). The data revealed NQO1 regulated the phosphorylation status of XIAP and the protein stability through AKT activation.

### SIRT6 involves in NQO1-mediated regulation of AKT signaling

It has been reported that deacetylation of AKT by sirtuin family members, which are class III NAD^+^-dependent protein deacetylation, is necessary for its activation [35, 36]. On the other hand, NQO1 catalyzes the reduction of quinones to hydroquinone by utilizing NADH as an electron donor, which consequently increases intracellular NAD^+^ levels [37]. Cellular NAD^+^ acts as a cofactor for many enzymes, particularly the sirtuins [38]. These finding prompted us to hypothesize that NQO1-mediated regulation of sirtuin members might account for AKT phosphorylation and activation in HCC cells. To test the hypothesize, we first examined expression of sirtuin members (SIRT1-SIRT7) in NQO1-depleted HCC cells by real-time PCR and immunoblotting analysis. Knock-down of NQO1 markedly resulted in decreased protein level of SIRT6, whereas it had no obvious effect on other sirtuin members (Additional file 1: Figure S1a-b). The total protein levels of AKT and phosphorylated AKT were analyzed in sirtuin members (SIRT1-SIRT7) knock-down cells. Interestingly, SIRT1, SIRT2 and SIRT6 suppression decreased phosphorylation of AKT without affecting total AKT level (Additional file 2: Figure S2). To this end, we focus on SIRT6 for further investigation.

### NQO1 increases XIAP phosphorylation via SIRT6/AKT axis

**Fig. 2:**
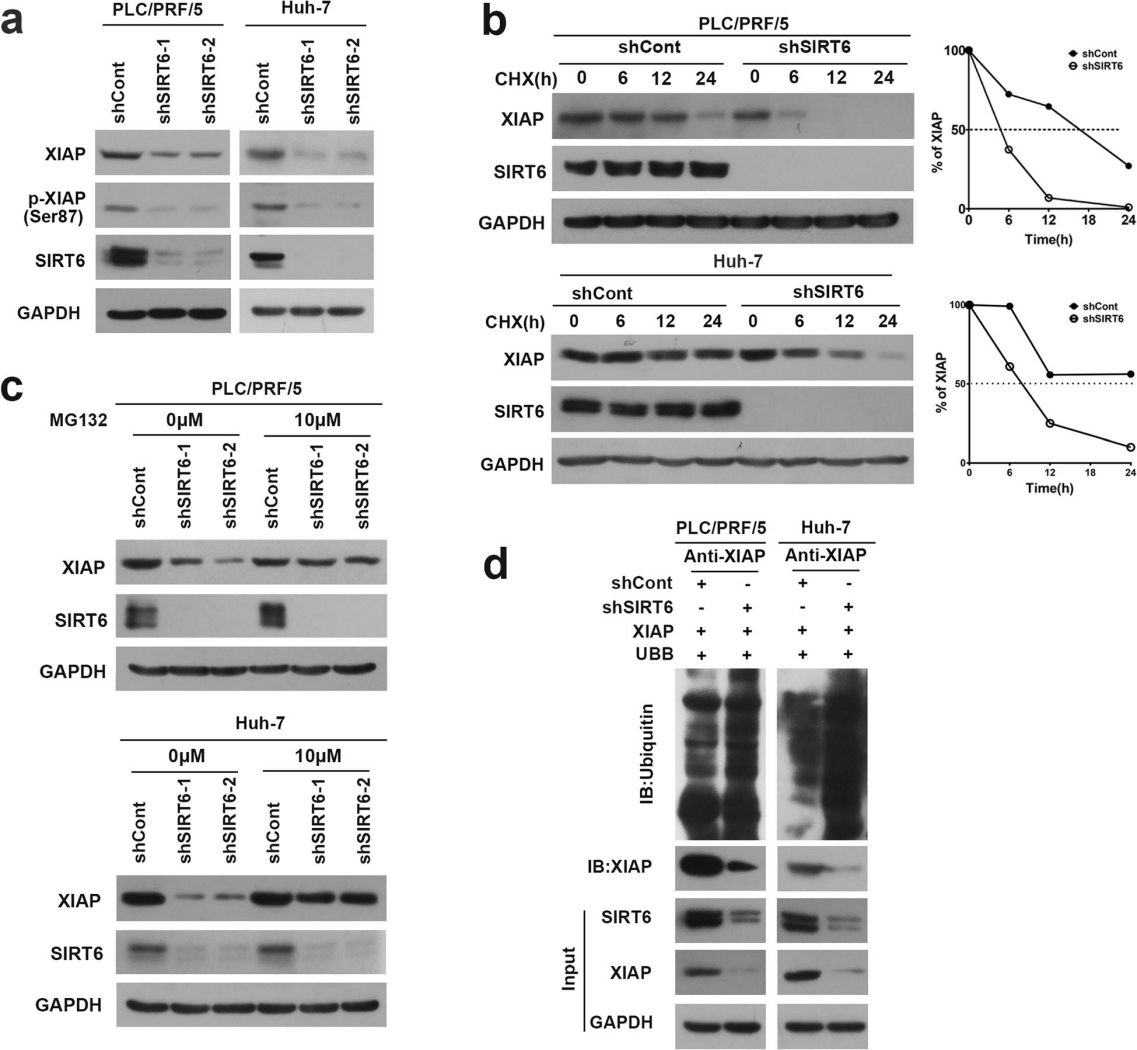
SIRT6 stabilizes XIAP protein from ubiquitin-mediated proteasome degradation. **a** Immunoblotting analysis for XIAP, phospho-XIAP (pSer87) and SIRT6 in SIRT6 knock-down cells. PLC/PRF/5 and Huh-7 cells were transduced with lentivirus expressing SIRT6-target shRNA (shSIRT6–1 and shSIRT6–2) and shCont. The expression of XIAP and phosphor-XIAP were analyzed 72 h after lentivirus transduction. **b** The protein levels of XIAP was examined by immunoblotting analysis. SIRT6 knock-down cells were incubated with 10 μg/mL cycloheximide and then harvested at the indicated times. **c** Immunoblotting analysis for XIAP in SIRT6 knock-down cells treated with proteasome inhibitor MG132 (10 μM) for 8 h. **d** XIAP ubiquitination were examined by immunoprecipitation assay. HA-Ub and XIAP were co-transfected into SIRT6 silencing or control cells; 72 h after transfection, cells were treated with MG132 for 8 h prior to be harvested to prevent proteasomal degradation

To identify whether XIAP is the downstream target of SIRT6, we first examined XIAP expression in SIRT6-depleted cells. SIRT6 knock-down markedly decreased the level of both XIAP protein and phosphorylated-XIAP without affecting its mRNA level (Fig. 2a, and Additional file 3: Figure S3). To examine the role of SIRT6 on XIAP protein stability, SIRT6-depleted cells were treated with cycloheximide. When compared to control cells, the half-life of XIAP protein was obviously reduced in SIRT6-depleted cells (Fig. 2b). Under the treatment of MG132, the influence of SIRT6 depletion on XIAP was blocked which revealed SIRT6 can stabilize XIAP protein through inhibiting proteasomal degradation (Fig. 2c). XIAP has been shown to possess E3 ubiquitin ligase activity and phosphorylation of XIAP could promote its autoubiquitination. Thus, we next examine the effect of SIRT6 on XIAP ubiquitination. XIAP ubiquitination was markedly enhanced in cells co-expressing XIAP, shSIRT6 and HA-tagged ubiquitin (HA-Ub) compared to levels of control cells (Fig. 2d). Collectively, these results indicated that SIRT6 stabilizes XIAP protein from ubiquitin-mediated proteasome degradation.

**Fig. 3:**
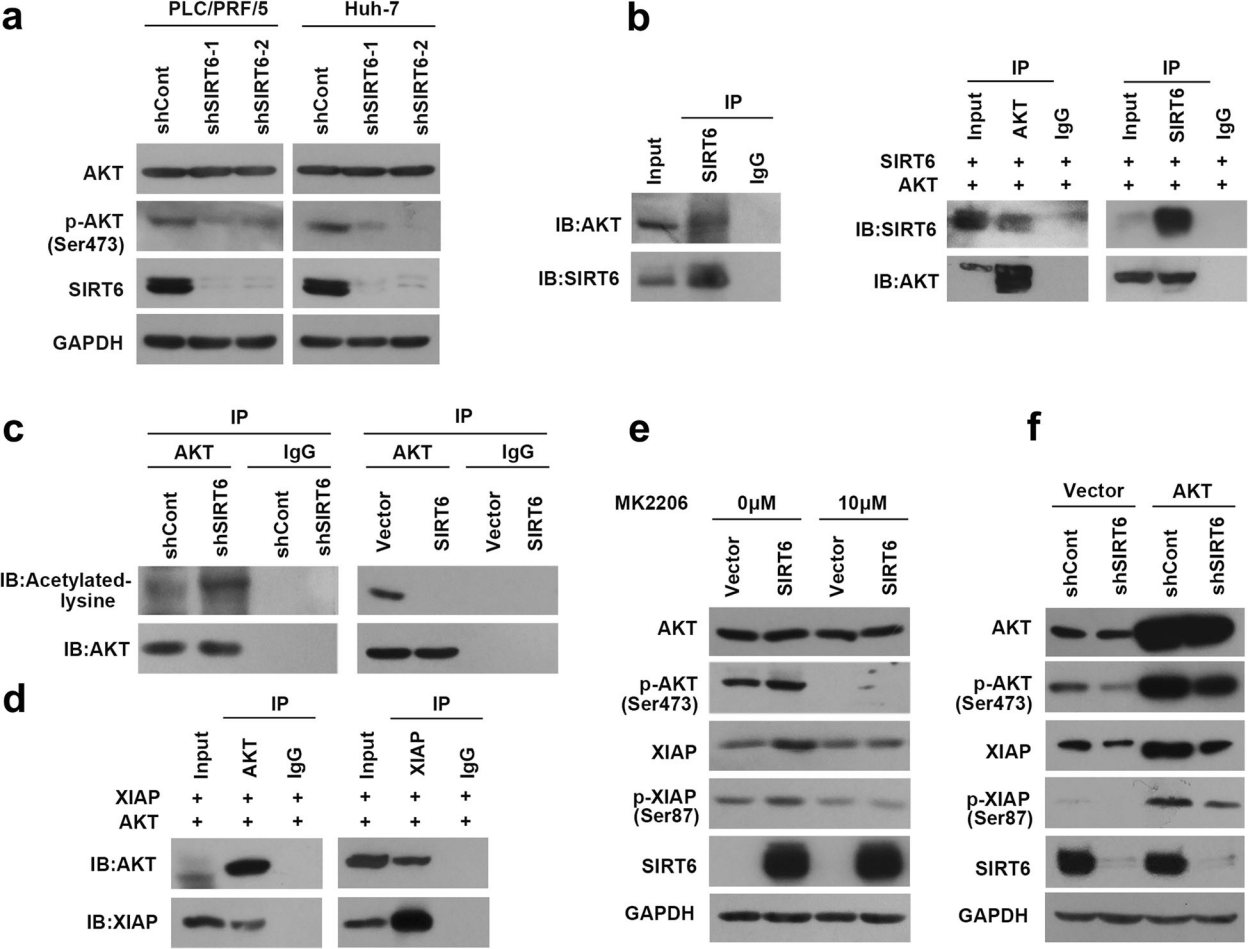
SIRT6 regulates phosphorylation and degradation of XIAP via deacetylation of AKT. **a** Immunoblotting analysis for AKT and phospho-AKT (pSer473) in SIRT6 knock-down cells. **b** Co-immunoprecipitation of AKT and SIRT6. Endogenous SIRT6 was immunoprecipitated using SIRT6 antibody and immunoblotted by AKT antibody (Left panel). The co-immunoprecipitation assay was also performed with the indicated antibodies in cells ectopically expressing SIRT6 and XIAP (Right panel). **c** The acetylation level of AKT was examined by immunoprecipitation assay with the anti-AKT antibody and probed with anti-acetylated-lysine antibody. **d** The interaction between AKT and XIAP was examined by the reciprocal co-immunoprecipitation in cells co-transfected with AKT and XIAP constructs. **e** Immunoblotting analysis for AKT, phosphor-AKT (pSer473), XIAP and phosphor-XIAP (pSer87) in SIRT6-overexpressing PLC/PRF/5 cells treated with AKT inhibitor MK2206 (10 μM) for 24 h. **f** Immunoblotting analysis for AKT, phosphor-AKT (pSer473), XIAP and phosphor-XIAP (pSer87) in SIRT6 knock-down cells transfected with AKT or empty vector

Next, we further investigated the regulatory role of SIRT6 on AKT. SIRT6 knock-down significantly decreased phosphorylation of AKT, whereas it did not affect total protein of AKT (Fig. 3a). The coimmunoprecipitation assay confirmed the endogenous SIRT6 was coprecipitation with endogenous AKT. This interaction was confirmed by ectopic expression of SIRT6 and AKT (Fig. 3b). SIRT6 silencing increased the acetylation level of AKT whereas SIRT6 overexpression exhibited opposite effect as determined by coimmunoprecipitation using anti-acetylation antibody (Fig. 3c). Moreover, immunoprecipitated AKT from HCC cells contained XIAP. Consistently, XIAP was coimmunoprecipitated with AKT (Fig. 3d). Importantly, AKT inhibitor (MK2206) remarkably inhibited SIRT6-induced increase of XIAP expression and phosphorylation (Fig. 3e). Introduction of AKT rescued SIRT6-silencing-induced decrease of XIAP expression and phosphorylation (Fig. 3f). Collectively, these data demonstrated SIRT6 regulated phosphorylation and degradation of XIAP via deacetylation of AKT.

### NQO1 stabilizes SIRT6 protein via blocking ubiquitin-mediated proteasome degradation

**Fig. 4:**
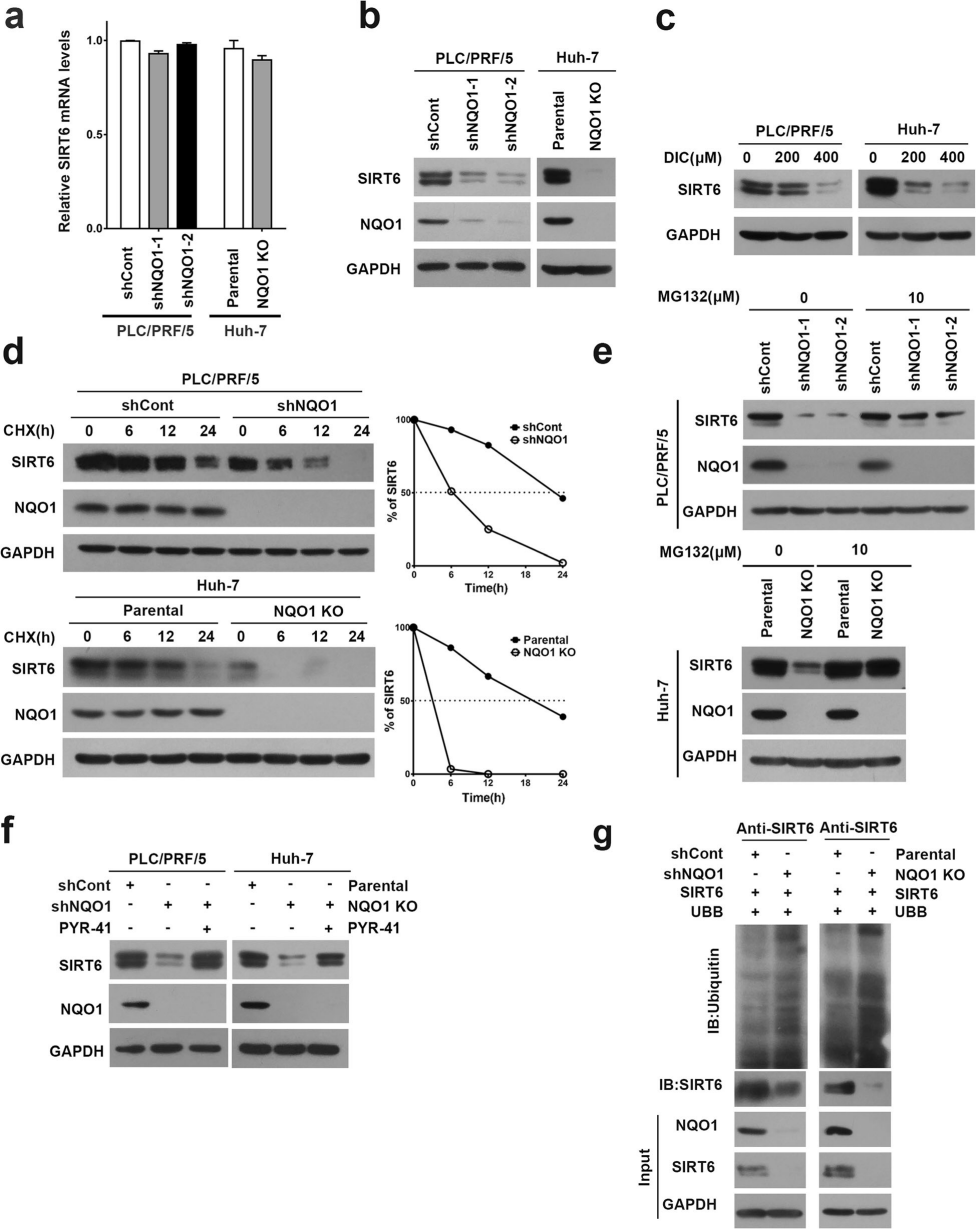
NQO1 knock-down/knock-out decreases the protein stability of SIRT6. **a** Real-time PCR for SIRT6 mRNA level in NQO1 knock-down PLC/PRF/5 and NQO1 knock-out Huh-7 cells. Data are mean ± SEM of *n* = 3 independent experiments. *p < 0.05. **b** Immunoblotting analysis for SIRT6 in NQO1 knock-down/knock-out cells. **c** Immunoblotting analysis for SIRT6 in PLC/PRF/5 and Huh-7 cells treated with dicoumarol (200 μM or 400 μM) for 4 h. **d** Immunoblotting analysis for SIRT6 in NQO1 knock-down/knock-out cells treated with 10 μg/ml cycloheximide at the indicated times. **e** Immunoblotting analysis for SIRT6 in NQO1 knock-down/knock-out cells treated with MG132 (10 μM) for 8 h. **f** Immunoblotting analysis of SIRT6 in NQO1-knock-down/knock-out cells treated with PYR-41 (50 μM) for 6 h. **g** SIRT6 ubiquitination was examined by immunoprecipitation assay in PLC/PRF/5 and Huh-7 cells

To study the potential mechanism of NQO1 regulates SIRT6, we first analyzed the mRNA and protein level of SIRT6 in NQO1-depleted or dicoumarol-treated cells. Consistently, loss-of-function of NQO1 (knock-down/knock-out or chemical inhibitor) resulted in reduced SIRT6 protein without affecting its mRNA level (Fig. 4a-c). To investigate whether NQO1 affected the stability of SIRT6 protein, we treated NQO1 knock-down cells with cycloheximide. When compared to control cells, the half-life of SIRT6 protein was obviously reduced in NQO1-depletion cells (Fig. 4d). The level of SIRT6 was restored in the presence of MG132 (Fig. 4e). These data supported the involvement of proteasomal machinery in NQO1 depletion-mediated degradation of SIRT6. To assess whether NQO1 stabilized SIRT6 via inhibiting ubiquitination, HCC cells were treated with PYR-41 (50 mM), a specific inhibitor of ubiquitin activating enzyme E1, thereby inhibiting whole-cell ubiquitination. PYR-41 treatment significantly restored the levels of SIRT6 protein in NQO1 depletion cells (Fig. 4f), suggesting that ubiquitination machinery was involved in NQO1 depletion-induced SIRT6 degradation. This was further confirmed by measuring the level of ubiquitinated-SIRT6 in NQO1 knock-down/knock-out cells. Loss-of-function of NQO1 increased the level of ubiquitination in SIRT6 (Fig. 4g).

**Fig. 5:**
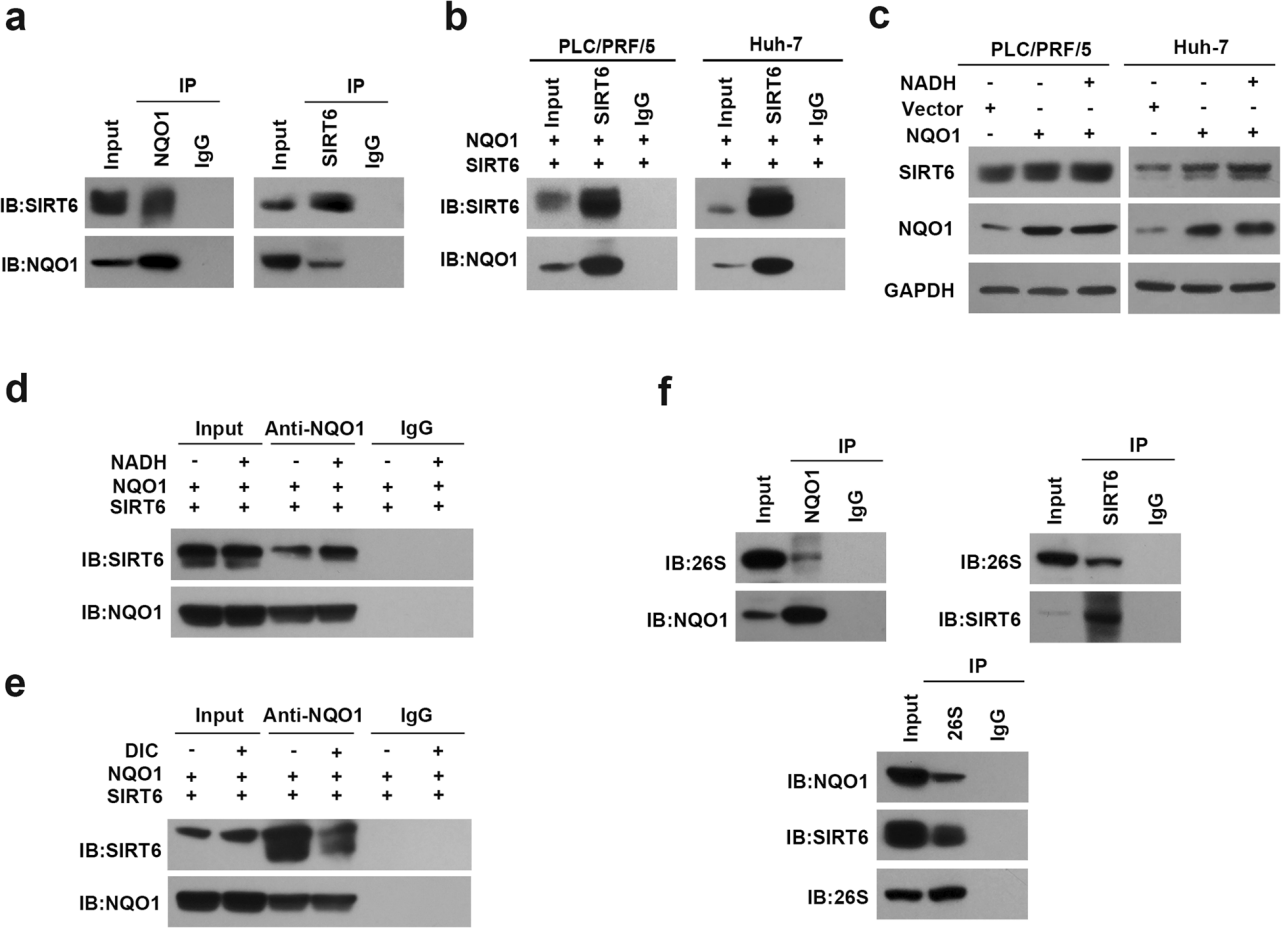
NQO1 stabilizes SIRT6 protein via inhibiting ubiquitin-mediated proteasome degradation. **a** Reciprocal co-immunoprecipitation of NQO1 and SIRT6. **b** The co-immunoprecipitation was performed in PLC/PRF/5 and Huh-7 cells co-transfected with NQO1 and SIRT6 constructs. **c** Immunoblotting analysis for SIRT6 in PLC/PRF/5 and Huh-7 cells ectopically expressing NQO1 treated with NADH (100 μM) for 4 h. **d**-**e** The interaction between NQO1 and SIRT6 was examined by co-immunoprecipitation assay. NQO1 and SIRT6 constructs were co-transfected into PLC/PRF/5 cells. At 48 h after transfection, cells were treated with NADH (100 μM) (**d**) and dicoumarol (400 μM) for 4 h (**e**). **f** Reciprocal co-immunoprecipitation assay was performed in PLC/PRF/5 cells using anti-NQO1, anti-SIRT6 and anti-26S antibody respectively, and immunoblotted with the indicated antibody

It has been reported that NQO1 stabilizes numerous proteins by physically binding to them and inhibiting ubiquitination [28, 39]. Therefore, we further identified whether NQO1 was physically related with SIRT6. Reciprocal co-immunoprecipitation assay with anti-NQO1 or anti-SIRT6 antibodies revealed the interaction between NQO1 and SIRT6 (Fig. 5a-b). On the other hand, NQO1 has been reported to regulate proteasomal degradation in a NADH-dependent manner [40]. Concordantly, we found that SIRT6 expression was remarkably increased in the presence of NQO1 cofactors NADH (Fig. 5c). Remarkable increase in the binding of NQO1 to SIRT6 was observed in the presence of NADH, whereas dicoumarol that competing with NADH for binding to NQO1 decrease the association between NQO1 and SIRT6 (Fig. 5d-e). These data indicated that NQO1 interacts physically with SIRT6 which requires binding to NADH.

Moreover, reciprocal co-immunoprecipitation assay revealed that NQO1 and SIRT6 were physically associated with 26S proteasome in HCC cells (Fig. 5f). These results indicated that NQO1 prevented SIRT6 from ubiquitin-mediated proteasome degradation. Furthermore, introduction of SIRT6 restored XIAP protein and rescued NQO1 depletion-inducing growth inhibition and apoptosis in HCC cells (Additional file 4: Figure S4). Collectively, these results demonstrated that NQO1 exerts its oncogenic function through stabilizing SIRT6, which consequently leads to AKT activation and XIAP phosphorylation.

### Reintroduction of SIRT6 rescues NQO1 depletion-induced apoptosis in mouse model

To study the oncogenic role of NQO1 in vivo, NQO1 knock-out cells with or without stable expression of SIRT6 or parental cell were orthotopically injected into livers. When compared to the parental cells, we verified that NQO1 knock-out significantly suppressed the HCC tumor sizes in orthotopically transplanted HCC mice.

**Fig. 6:**
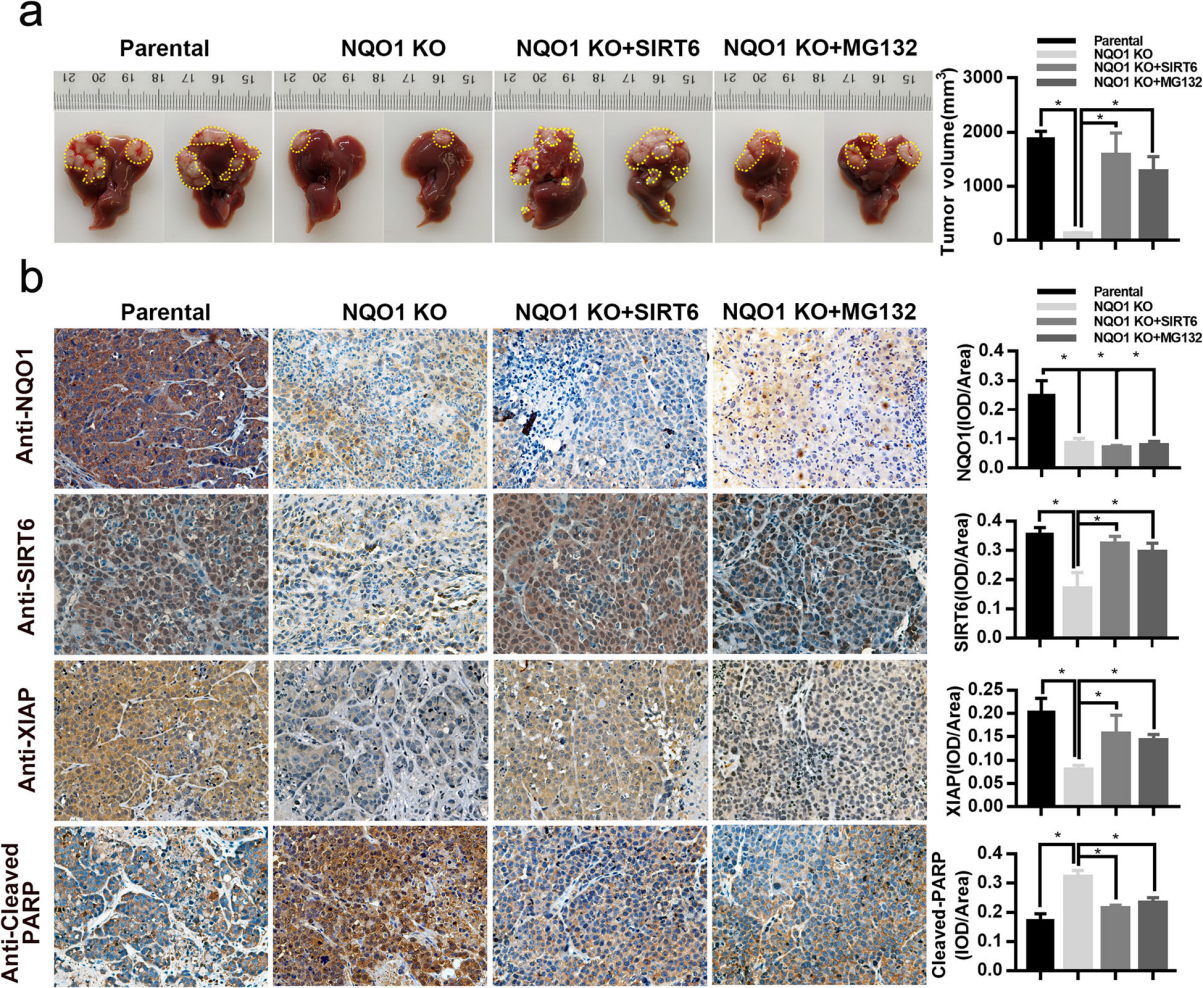
Reintroduction of SIRT6 antagonizes NQO1 deficiency-induced apoptosis in vivo. NQO1 knock-out cells Huh-7 cells, parental cells or NQO1 knock-out cells stably overexpressing SIRT6 were injected orthotopically into 4-week-old nude mice. At 5 weeks after implantation, the animals were sacrificed, and the tumor masses were excised. NQO1 knock-out groups were injected with or without MG132 (0.05 mg/kg, intraperitoneally, daily) for 30 days starting 5 days after the injection of HCC cells. **a** Representative images of tumors formed (Left panel) and tumor volume in different groups of nude mice (Right panel). **b** Representative images of immunohistochemical staining of NQO1, SIRT6, XIAP and Cleaved PARP in tumor xenografts counterstaining with hematoxylin. Magnification, × 400

Meanwhile, SIRT6 overexpression rescued the effect of NQO1 knock-out-mediated growth inhibitory (Fig. 6a). To investigate whether ubiquitin-proteasome pathway was involved in NQO1 depletion-induced apoptosis in vivo, NQO1 knock out groups were received injections with or without MG132. As shown in Fig. 6a, MG132 treatment blocked tumor growth inhibition induced by NQO1 knock out. Furthermore, IHC analysis showed that NQO1 knock-out was related with reduced level of SIRT6, XIAP and increased level of cleaved PARP in tumor tissues. Importantly, the above effects could be reversed by SIRT6 overexpression or MG132 treatment partially (Fig. 6b). These data suggested that MG132 attenuates NQO1 knock out-induced downregulation of SIRT6 and activation of SIRT6-XIAP axis.

## Discussion

As shown in previous studies, NQO1 overexpression in HCC cells enhanced XIAP protein stability through promoting its phosphorylation [8], which determined its activity of autoubiquitination/ubiquitination and protein stability [41, 42]. In this manuscript, we investigated the underlying mechanism how NQO1 regulated XIAP phosphorylation. It has been reported that the phosphorylation status at Serine 87 of XIAP could be regulated by AKT protecting it from ubiquitination and degradation [34, 41], which prompting us to hypothesize that in our model XIAP may be a downstream target of AKT. Consistent with previous studies, our data suggested loss-of-function of NQO1 reduced the phosphorylated status of AKT without affecting total AKT expression, whereas NQO1 overexpression increased level of phosphorylated AKT. Importantly, AKT inhibitor (MK2206) remarkably blocked NQO1-induced increase of XIAP protein and phosphorylation level, whereas increased phosphorylated AKT restored XIAP protein and phosphorylation level in NQO1-deleted cells. These data suggested NQO1 could affect AKT phosphorylation and activation to promote XIAP phosphorylation and stability in HCC cells.

Acetylation is one of mechanisms participated in the direct or indirect regulation of AKT activation [35, 43, 44]. Earlier studies have reported that deacetylation of AKT by SIRT1, SIRT2 and SIRT7, which are class III NAD^+^-dependent protein deacetylases, is necessary for its activation [35, 36]. SIRT1 deacetylated the two conserved lysine residues of AKT PH domain, contributing to the binding of AKT to PIP3, and thereby increasing membrane localization and activation [35]. Our previous study has revealed SIRT2 regulated the deacetylation and activation of AKT, which subsequently activated GSK3β/β-catenin-signaling axis to regulate EMT in HCC [36]. Additionally, SIRT7 was also reported to regulate FKBP51 acetylation and enhances complex formation of FKBP51-AKT-PHLPP. This subsequently suppressed AKT pathway and then increased chemo-sensitivity in breast cancer cell [43]. Consistently, our present data demonstrated SIRT6 as downstream protein of NQO1 which was responsible for AKT activation by screening sirtuin members. Mechanistically, SIRT6 deacetylated and activated AKT, thus leading to increased XIAP phosphorylation and protein stability in HCC cells. However, our finding was inconsistent with previously published results that SIRT6 overexpression negatively regulated PI3K signaling pathway without affecting AKT phosphorylation. It was on account of SIRT6 exerted anti-tumor sphere-forming at the transcriptional level which was independent of SIRT6 histone deacetylase activity [45]. The contradictory of different function of SIRT6 in these studies may be result from tissue-specific molecular profiles, the context and gene dose.

NQO1 exercises a selective “gatekeeping” role in regulating the proteasomal degradation of specific proteins. NQO1 binds to and thereby stabilizes the important tumor suppressor p53 against 20S proteasomal degradation [46]. In addition to p53, NQO1 also regulates the ubiquitin-independent 20S proteasomal degradation of p73α and p33 [23, 40]. Downregulation of NQO1 by HIV Rev. induces ubiquitin-independent proteasomal degradation of Tat, which is implicated in HIV-gene expression and latency [47]. But recently, Oh *et.al* has revealed that NQO1 stabilizes HIF-1α by inhibiting the level of ubiquitination and the 26S proteasomal degradation [28]. Consistent with Oh *et.al,* we found that both NQO1 and SIRT6 were physically associated with 26S proteasomes in HCC cells, suggesting that NQO1 stabilizes SIRT6 by blocking ubiquitination-dependent proteasomal degradation. This finding was further confirmed in vivo. MG132 treatment blocked tumor growth inhibition induced by NQO1 knock out, accompanied with increased level of SIRT6 and XIAP. MG132, which acts as a blocker in ubiquitin-proteasome pathway, is involved in > 80% of intracellular protein degradation. However, its role in apoptosis of cancer cell is controversial. MG132 promotes the cisplatin-induced apoptosis and inhibits tumor growth [48, 49], however, it blocks high-dose UV irradiation-induced apoptosis [50]. Additionally, MG132 also blocks bufalin-induced cell apoptosis by preventing the degradation of anti-apoptotic Bcl-2 family member (Mcl-1) [51]. In this study, MG132 treatment blocks NQO1 depletion-induced apoptosis, supporting its role in inhibiting apoptosis. Basing on our current data, we suggest that NQO1 binds to SIRT6 and inhibits ubiquitin-dependent proteasomal degradation.

## Conclusions

**Fig. 7:**
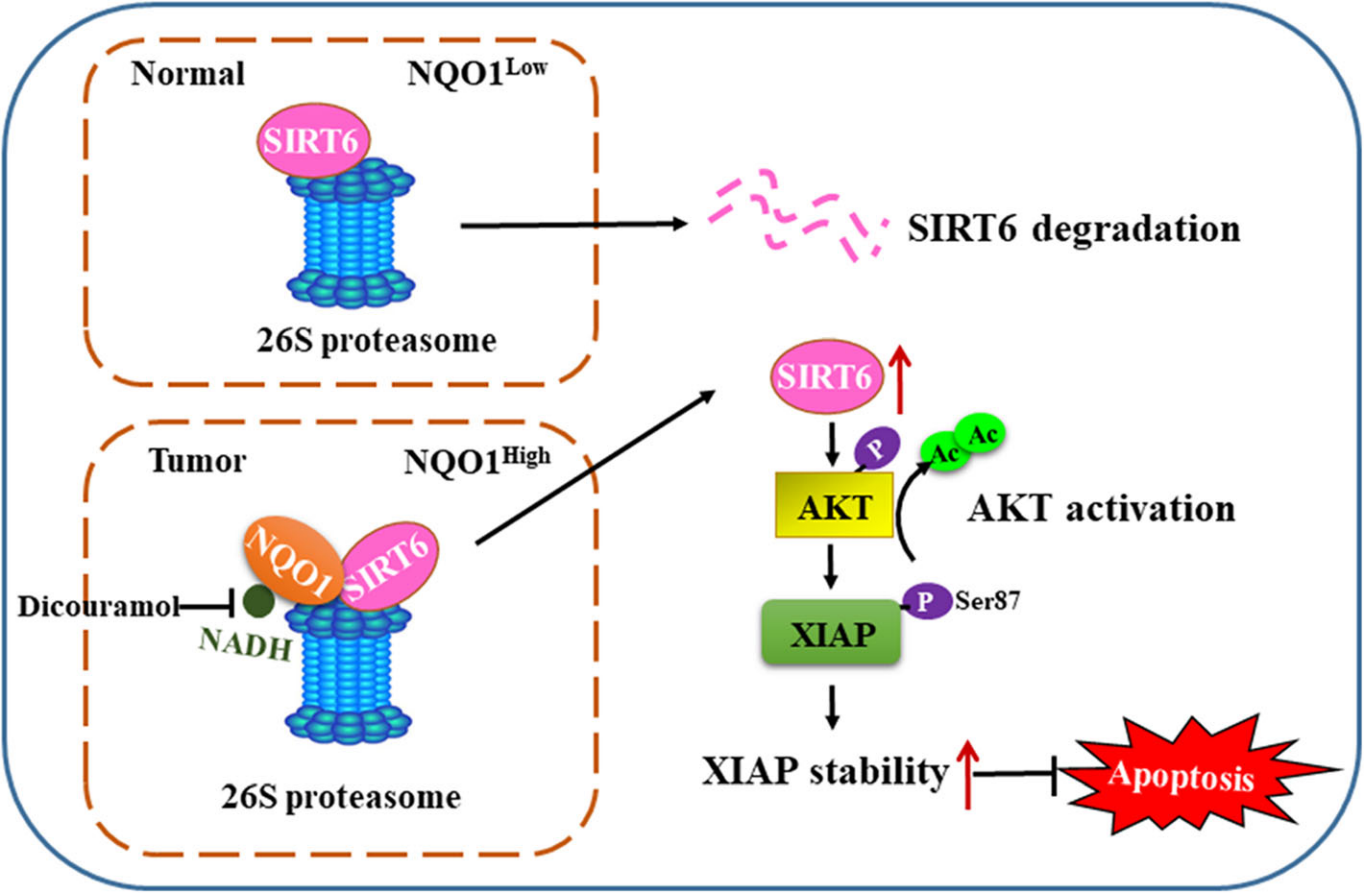
Schematic model of how NQO1 inhibits HCC apoptosis. The working model for oncogenic role of NQO1 in HCC. In HCC cells where NQO1 expression is high, NQO1 interacts physically with SIRT6, stabilizes the protein and prevents it from ubiquitin-dependent proteasomal degradation. Consequently, SIRT6 deacetylated AKT to promote its phosphorylation and activation, thus leading to increasing XIAP phosphorylation and protein stability

In summary, our findings determined NQO1 exerts its oncogenic function by regulating SIRT6/AKT/XIAP pathway. In HCC cells where NQO1 expression is high, NQO1 physically interacts with SIRT6 and stabilizes its protein against ubiquitin-dependent proteasomal degradation. Consequently, SIRT6 deacetylated AKT to promote its phosphorylation and activation, thus leading to increase XIAP phosphorylation and protein stability (Fig. 7). Our findings provide insights on the meaning of SIRT6/AKT/XIAP axis for NQO1-mediated tumorigenesis.

## Supplementary information

**Additional file 1: Figure S1.** Effect of NQO1 silencing on seven sirtuin members. (a) Real-time PCR for SIRT1–7 mRNA level in NQO1 knock-down PLC/PRF/5 cells. Data are mean ± SEM of *n* = 3 independent experiments. (b) Immunoblotting analysis for SIRT1–7 in NQO1-silencing cells.

**Additional file 2: Figure S2.** Effect of sirtuin family members silencing on AKT. Immunoblotting analysis for total AKT and phospho-AKT in sirtuin members (SIRT1-SIRT7) knock-down cells.

**Additional file 3: Figure S3.** Effect of SIRT6 silencing on XIAP. Real-time PCR for XIAP mRNA level in SIRT6 knock-down PLC/PRF/5 and Huh-7 cells. Data are mean ± SEM of *n* = 3 independent experiments.

**Additional file 4: Figure S4.** Ectopic expression SIRT6 antagonizes the effect of NQO1 deficiency on proliferation and apoptosis. (a) Immunoblotting analysis for NQO1, SIRT6, XIAP and Cleaved-PARP in NQO1-depleted PLC/PRF/5 cells transfected with vector expressing SIRT6. (b) Trypan blue exclusion assay for NQO1-depleted PLC/PRF/5 cells or control cells transfected with vector expressing SIRT6. (c) Flow cytometry with Annexin V/Pi for NQO1-depleted PLC/PRF/5 cells transfected with vector expressing SIRT6.

## Data Availability

All data used in this study are fully available without restrictions.

## Abbreviations

CHX: Cycloheximide
DIC: Dicoumarol
HCC: Hepatocellular Carcinoma
NAD: Nicotinamide adenine dinucleotide
NADH: Nicotinamide adenine dinucleotide plus hydrogen
NADPH: Nicotinamide adenine dinucleotide phosphate plus hydrogen
NQO1: NAD(P)H: quinone oxidoreductase 1
SIRT6: Sirtuin 6
UBB: Ubiquitin B gene
XIAP: X-linked inhibitor of apoptosis protein
