# Supplementary figures and images for: NQO1 potentiates apoptosis evasion and upregulates XIAP via inhibiting proteasome-mediated degradation SIRT6 in hepatocellular carcinoma
Source: Cell Commun Signal. 2019 Dec 16;17:168. doi: 10.1186/s12964-019-0491-7 (PMC6915971; doi:10.1186/s12964-019-0491-7)

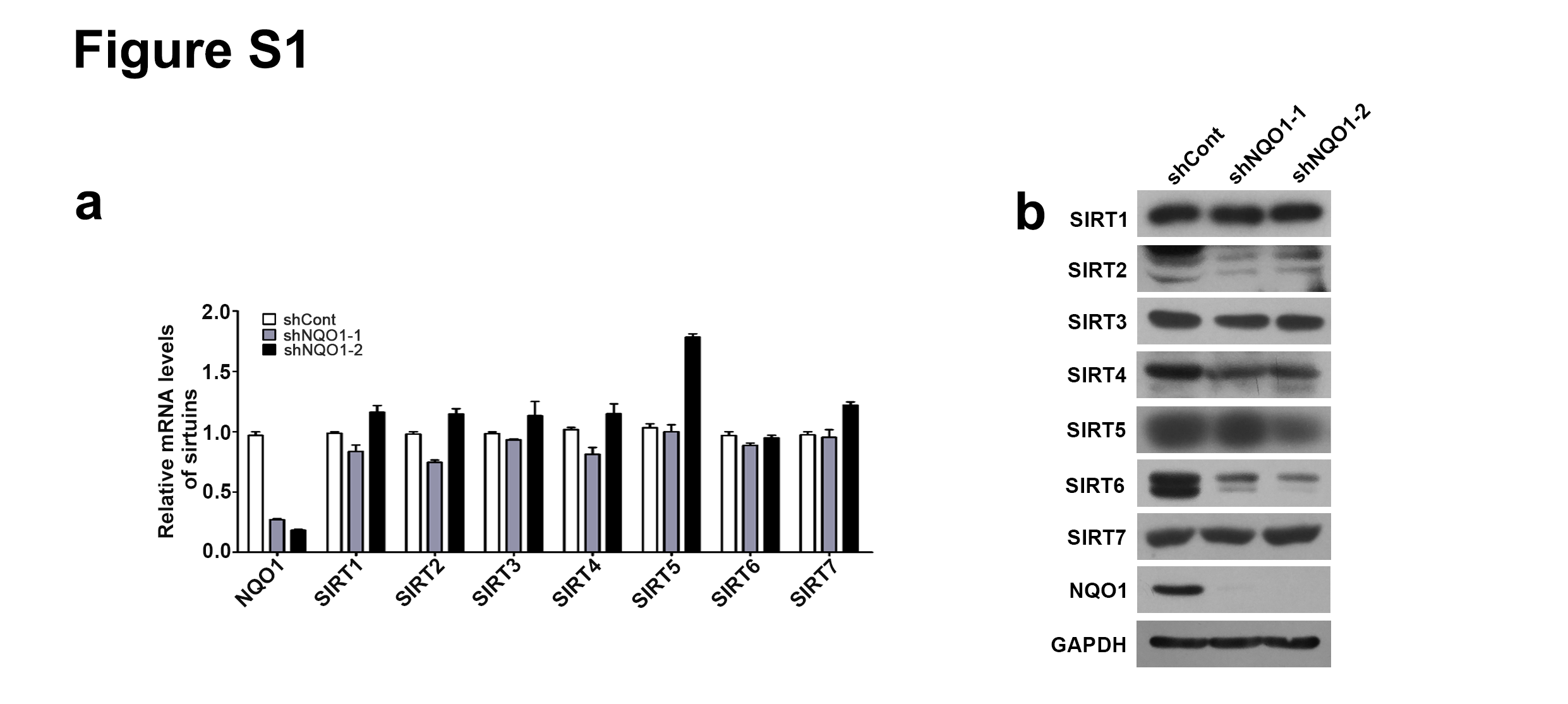

Supplement: Supplementary file 1 — Additional file 1: Figure S1. Effect of NQO1 silencing on seven sirtuin members. (a) Real-time PCR for SIRT1–7 mRNA level in NQO1 knock-down PLC/PRF/5 cells. Data are mean ± SEM of n = 3 independent experiments. (b) Immunoblotting analysis for SIRT1–7 in NQO1-silencing cells. [file 12964_2019_491_MOESM1_ESM.tif]

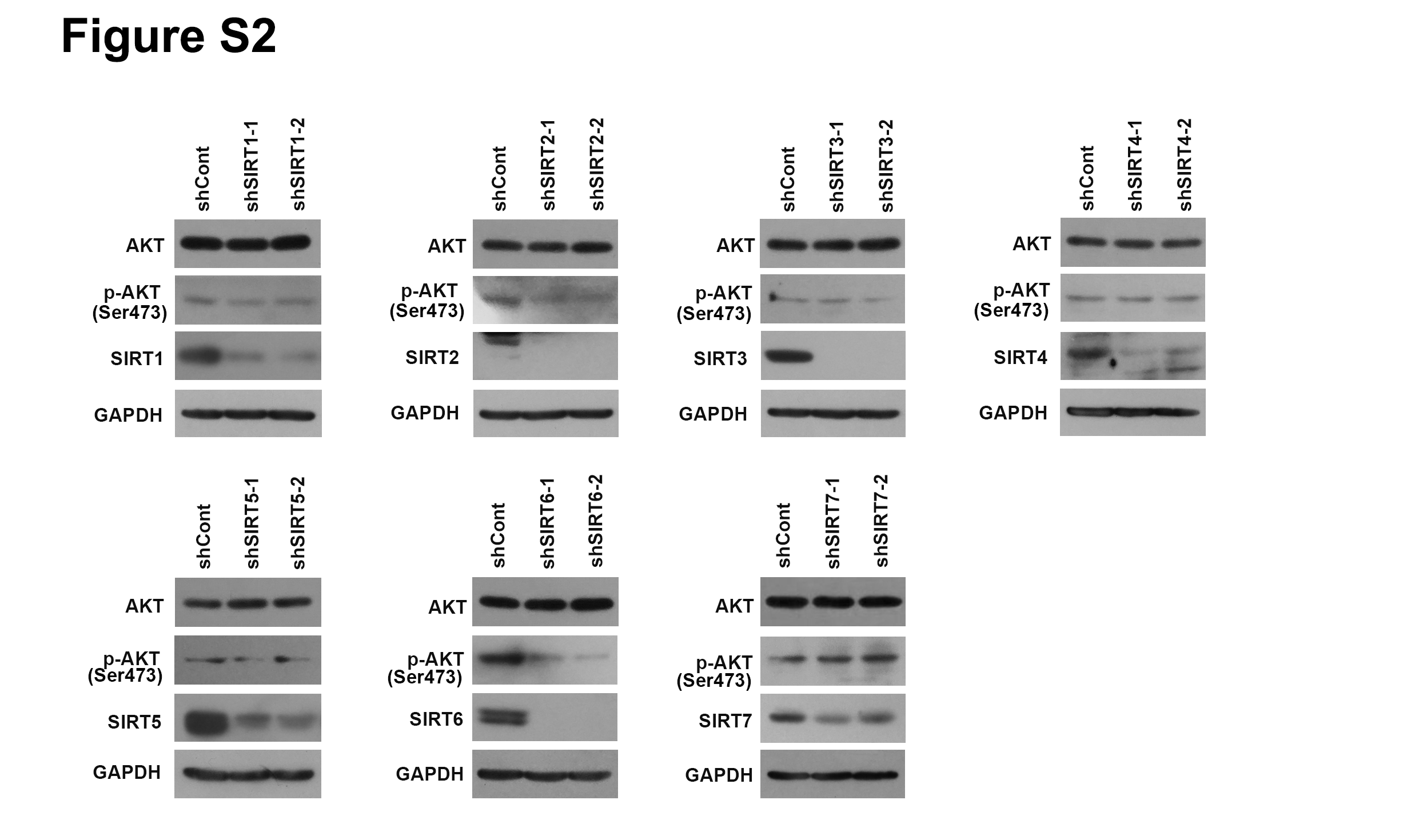

Supplement: Supplementary file 2 — Additional file 2: Figure S2. Effect of sirtuin family members silencing on AKT. Immunoblotting analysis for total AKT and phospho-AKT in sirtuin members (SIRT1-SIRT7) knock-down cells. [file 12964_2019_491_MOESM2_ESM.tif]

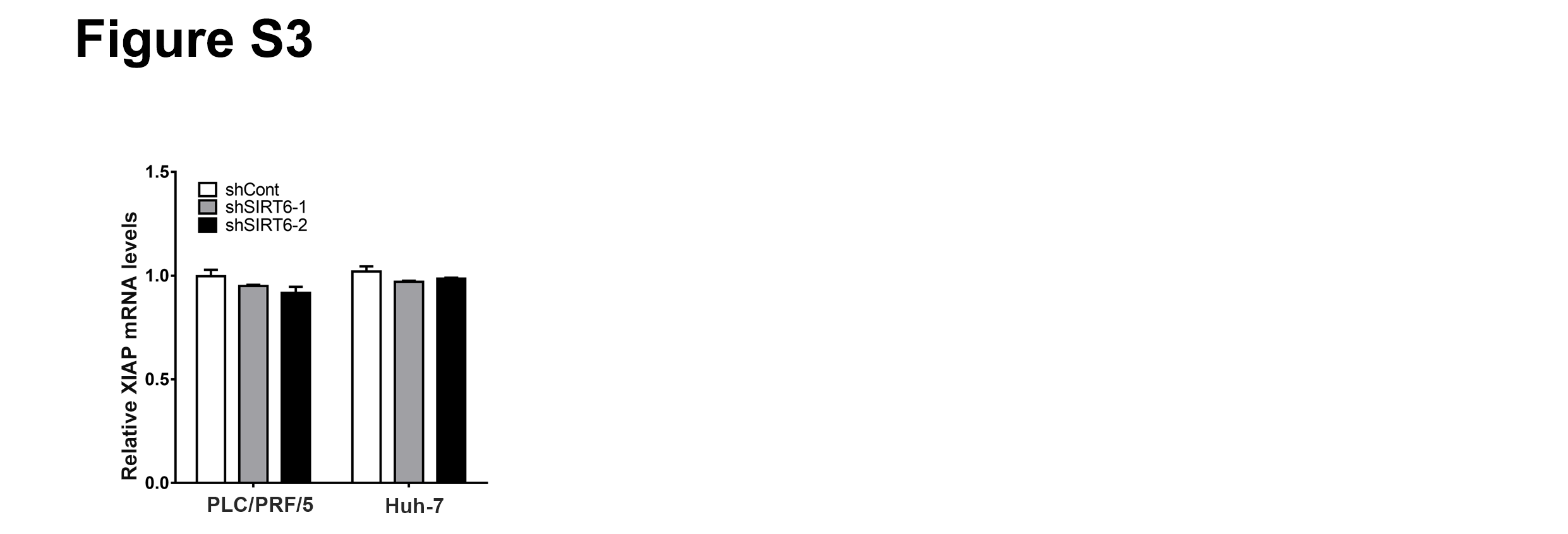

Supplement: Supplementary file 3 — Additional file 3: Figure S3. Effect of SIRT6 silencing on XIAP. Real-time PCR for XIAP mRNA level in SIRT6 knock-down PLC/PRF/5 and Huh-7 cells. Data are mean ± SEM of n = 3 independent experiments. [file 12964_2019_491_MOESM3_ESM.tif]

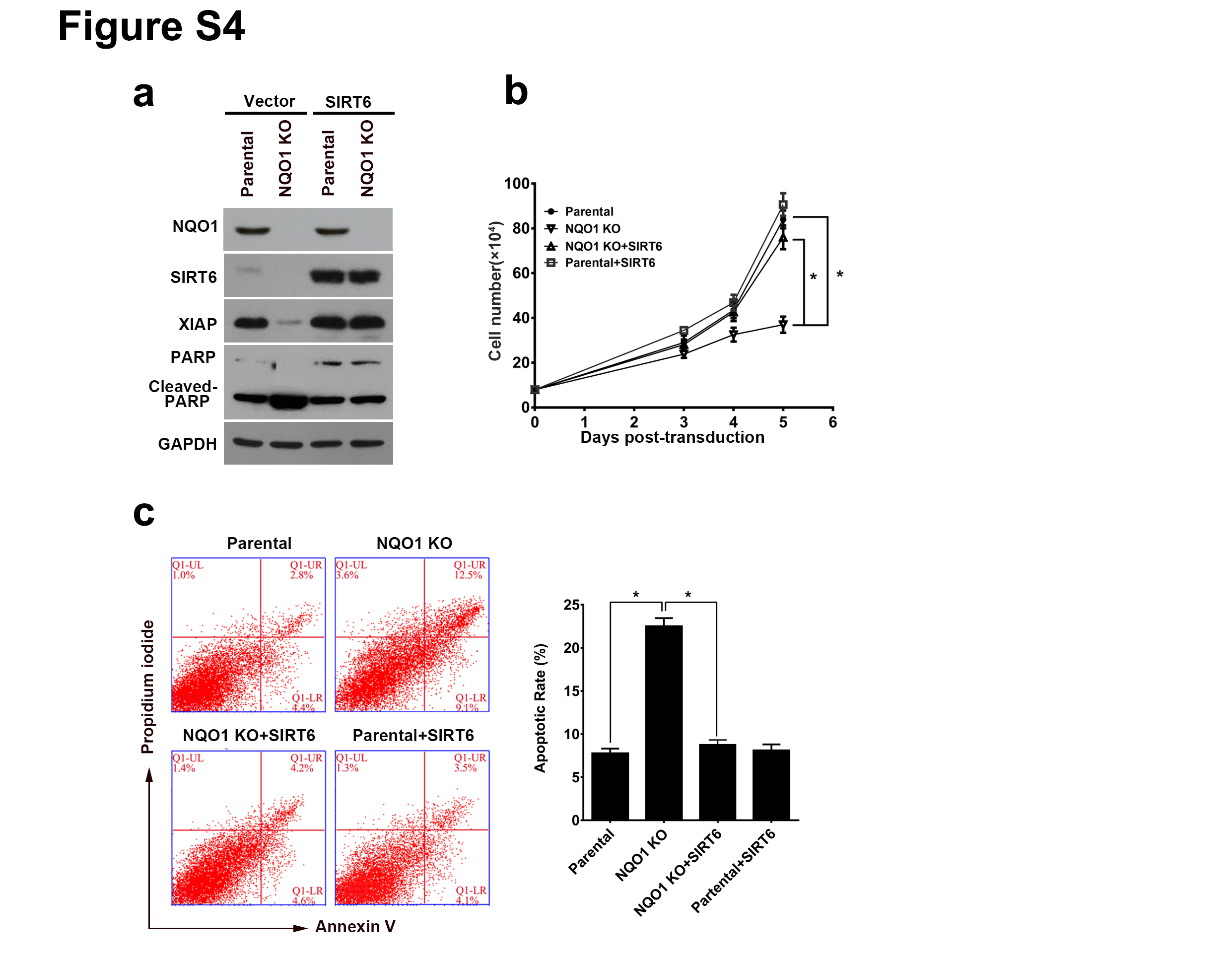

Supplement: Supplementary file 4 — Additional file 4: Figure S4. Ectopic expression SIRT6 antagonizes the effect of NQO1 deficiency on proliferation and apoptosis. (a) Immunoblotting analysis for NQO1, SIRT6, XIAP and Cleaved-PARP in NQO1-depleted PLC/PRF/5 cells transfected with vector expressing SIRT6. (b) Trypan blue exclusion assay for NQO1-depleted PLC/PRF/5 cells or control cells transfected with vector expressing SIRT6. (c) Flow cytometry with Annexin V/Pi for NQO1-depleted PLC/PRF/5 cells transfected with vector expressing SIRT6. [file 12964_2019_491_MOESM4_ESM.tif]
